# Breaking paradigms: Long non-coding RNAs forming gene fusions with potential implications in cancer

**DOI:** 10.1016/j.gendis.2023.101136

**Published:** 2023-10-11

**Authors:** David Sánchez-Marín, Macrina Beatriz Silva-Cázares, Fany Iris Porras-Reyes, Rebeca García-Román, Alma D. Campos-Parra

**Affiliations:** a Posgrado en Ciencias Biológicas, Facultad de Medicina, Universidad Nacional Autónoma de México, Ciudad de México, C.P. 04360, México; b Unidad Académica Multidisciplinaria Región Altiplano, Universidad Autónoma de San Luis Potosí (UASLP), Carretera a Cedral Km 5+600, Ejido San José de la Trojes, Matehuala, San Luis Potosí, C.P. 78760, México; c Servicio de Anatomía Patológica, Instituto Nacional de Cancerología (INCan), Niño Jesús, Tlalpan, Ciudad de México, C.P. 14080, México; d Instituto de Salud Pública, Universidad Veracruzana (UV), Av. Dr Luis, Dr. Castelazo Ayala s/n, Col. Industrial Ánimas, Xalapa, Veracruz, C.P. 91190, México

**Keywords:** Cancer, Gene fusions, lncRNAs, ncRNAs, Therapy

## Abstract

Long non-coding RNAs (lncRNAs) are non-coding RNAs longer than 200 nucleotides with dynamic regulatory functions. They interact with a wide range of molecules such as DNA, RNA, and proteins to modulate diverse cellular functions through several mechanisms and, if deregulated, they can lead to cancer development and progression. Recently, it has been described that lncRNAs are susceptible to form gene fusions with mRNAs or other lncRNAs, breaking the paradigm of gene fusions consisting mainly of protein-coding genes. However, their biological significance in the tumor phenotype is still uncertain. Therefore, their recent identification opens a new line of research to study their biological role in tumorigenesis, and their potential as biomarkers with clinical relevance or as therapeutic targets. The present study aimed to review the lncRNA fusions identified so far and to know which of them have been associated with a potential function. We address the current challenges to deepen their study as well as the reasons why they represent a future therapeutic window in cancer.

## Introduction

Cancer is a genetic disease characterized by multiple changes in the genome. Among the genomic structural variations are gene fusions, which have gained relevance as it is estimated that 20% of cancer morbidity is due to their occurrence.^1^ Gene fusions are hybrid genes formed by two originally independent genes derived from inversions, deletions, translocations, tandem repeats, or trans/cis splicing of pre-mRNAs.^2^ Their contribution to cancer development is due to loss or gain of function. Studies have only focused on identifying protein-coding gene (PCG) fusions, most of which remain functionally uncharacterized.^3^

Long non-coding RNAs (lncRNAs) have gained great interest in their field of study because they are extremely versatile molecules in terms of function and localization.^4^, ^5^, ^6^ LncRNAs are defined as non-coding RNAs (ncRNAs) with a length greater than 200 nucleotides that are not translated into functional proteins due to the nonexistence of an open reading frame (ORF), although some of them can be translated into small functional peptides.^7^^,^^8^ They are capable of binding to several molecules by base complementary or structure recognition since they fold in three-dimensional secondary structures, which allows their interaction with other lncRNAs, microRNAs, mRNAs, proteins, chromatin, and, even phospholipids, to regulate numerous cellular processes.^9^, ^10^, ^11^ Hence, lncRNAs are dynamic regulatory molecules thanks to the multiple interactions they sustain as well as their localization in several cellular sites such as the nucleus, cytoplasm, and mitochondria.^4^^,^^12^ Their dysregulated expression, mutations, single nucleotide polymorphisms (SNP), and the recently identified lncRNAs fusions have been associated with cancer. Like PCG, lncRNAs can also function as both, tumor suppressors or oncogenes. Moreover, it has also been reported that they can regulate signaling pathways, the interconnection between them, and even mediate their expression through feedbacks regulating proliferation, survival, invasion, metastasis, and resistance to anticancer drugs.^13^ Strikingly, in addition to the deregulation of signaling pathways caused by the generation of a gene fusion per se, the generation of a gene fusion also causes changes by altering the dysregulation or malfunction of the involved genes.^14^ As described below, several genomic alterations in coding and non-coding regions are of interest.

## Genomic alterations of lncRNAs in cancer

Genomic alterations have been identified in almost every gene, and in some cases, it has been determined that they can contribute to disease development such as cancer. Significant advances in personalized medicine have been possible by identifying mutations, enabling targeted therapy against specific proteins carrying such mutations, which would otherwise not be effective.^15^^,^^16^ Nonetheless, current studies describing gene fusions have a PCG center-view as the effect is on the function. It is likely for gene fusions of PCG to present a deleterious effect such as a deletion, insertion, translocation, or frameshift alteration. Besides, it has been easier to describe the effect of genomic alterations in exons rather than introns, and that is the reason for which most mutations in this region remain as variants of unknown clinical significance.^17^ It was previously emphasized that the structure of lncRNAs is crucial in maintaining interactions through several conformations for their biological roles, hence, genomic alterations in the molecules can also affect their function despite most of them not being translated to proteins.

Efforts have been made to elucidate possible functional roles of variants in lncRNAs genomic regions. For instance, a recent study pointed out a panel of 8 well-studied lncRNAs with 10 driver mutations from a lncRNA 112 census with known roles in cancer: DLEU2, GAS5, MONC, NEAT1, PINT, PVT1, SLNCR1, and XIST.^18^ These mutations confer either an oncogene or tumor suppressor gene activity. Among these, NEAT1 has recurrent mutations in breast cancer, however, it is still not clear the mechanisms responsible for its oncogenic role. On the contrary, PVT1 is a highly expressed lncRNA known for contributing to tumorigenesis, as it is in a fragile region of chromosome 8, it is amplified and co-transcribed with adjacent oncogene c-Myc.^19^ In parallel, PVT1 amplification causes the stabilization of KLF5 and STAT3 oncoproteins.^20^^,^^21^ Evidence shows that, in cancer, lncRNA genomic alterations have functional repercussions as they not only change the sequence but they are subject to structural and epigenetic alterations. Interestingly, MALAT1 has not been associated with the lncRNA driver mutation panel despite being one of the most susceptible genes to mutations in cancer. Nonetheless, mutations in MALAT1 can impair its m^6^A methylation, causing the triple helix structure not to form. In turn, the lncRNA is degraded and its function is impaired.^22^ Singh, B. identified several significantly mutated regions in non-coding regions such as introns, untranslated regions, and lncRNAs. Significantly mutated regions are in motifs of RNA-binding proteins like SRSF10 and MBNL1, a finding that represents the importance of mutations in lncRNAs as they exert regulatory functions at the transcriptional level in cis and trans.^23^

Likewise, alterations described so far are related to large rearrangement or other deleterious mutations, SNPs in both coding and non-coding genes have also been associated with cancer risk.^24^ Interestingly, SNPs are wide events in non-coding RNAs. A recent study described the prevalence of 495,729 SNPs across 17,436 lncRNAs.^25^ The first SNPs were detected in HOTAIR and H19 lncRNAs. In H19, the SNP Rs217727 C/T has been reported in exons affecting the three-dimensional structure of the lncRNA. However, as this topic has been little explored, up to date it is only known that this SNP is associated with breast cancer susceptibility and it remains unexplored if the SNP is located within a sequence that interacts with other molecules.^26^^,^^27^ Regarding HOTAIR SNPs, ten SNPs have been reported to affect the interaction with different transcription factors (EZH2, CTBP2, CHD1, ZNF143, SUZ12, TCF7L2, CTCF, RAD21, and YY1) due to an impact on their structure. Mainly SNPs rs920778 and rs7958904 may favor the development of cancer.^28^ Recently, a meta-analysis showed that the rs920778, rs4759314, rs874945, and rs12826786 polymorphisms in HOTAIR increase sensitivity to several types of cancer.^29^

Some studies describe the relationship between SNPs and the regulation of lncRNAs. At the transcriptional level, an SNP up-regulates an oncogene, GAS5, by altering its methylation status in colorectal cancer and hepatocarcinoma.^30^ On the contrary the tumor suppressor lncRNA PTCSC3 is down-regulated as an SNP diminishes its binding to transcriptional activators such as C/EBPα and C/EBPβ.^31^ At the post-transcriptional level, an SNP in LINC00673 creates a microRNA binding site for miR-1231, which converts the lncRNA into a target of miR-1231.^32^ Other well-known oncogene lncRNAs with reported SNPs (*e.g.*, ANRIL, H19, and PCGEM1) are associated with an increased risk of cancer and elevated expression compared with the wild type version of each gene, and interestingly, the AQP4-AS1 lncRNA SNP is specific of a Brazilian cohort.^33^, ^34^, ^35^, ^36^ Furthermore, SNPs in lncRNAs can impact the cell phenotype. For example, an SNP in the 1q41 locus creates a binding motif of BAT, altering the enhancer activity, and thus up regulating lncSLCC1. Subsequently, this lncRNA transcriptionally activates HK2, a crucial enzyme in glucose metabolism, promoting tumor growth in colorectal cancer.^37^

All these studies strongly suggest that genomic alteration in non-coding regions, even as small as SNPs, can both, promote tumorigenesis through altering their regulatory function based on their structure, or trigger other genomic and epigenetic alterations that contribute to cancer development. Either of them, the molecular mechanisms remain unknown and more work must be done to elucidate them. Among the gene alterations that have been well described and even used as therapeutic targets, coding gene fusions stand out as one such alteration.

## Gene fusions involving PCG: highlights in cancer

Gene fusion might be the most impressive resource employed by tumor cells to produce genomic diversity with unique functions that enhance cancer development. Gene fusions are the resulting structural rearrangements of the genome such as inversions, deletions, tandem repeats, trans/cis splicing of pre-mRNAs, or translocations producing the fusion of two unrelated genes.^2^ The first gene fusion reported was the BCR/ABL gene in leukemias. It is formed by a reciprocal chromosomal translocation generating a fusion protein with a constitutively active kinase domain that allows it to signal all the time by activating cancer-promoting signaling pathways.^38^ Afterward, other gene fusions were soon identified in solid tumors. For instance, the oncogenic EML4–ALK fusion, resulting from the inversion of chromosome 2, on which both genes are located, was described in anaplastic lymphoma, and later in patients with non-small cell lung cancer. This inversion produces a protein with the tyrosine kinase region of ALK and the amino-terminal end of EML4 capable of dimerizing and activating downstream signaling pathways.^39^ TMPRSS2–ERG fusion protein, caused by chromosome deletion, is the most common fusion in prostate cancer. It mediates the overexpression of E26 transformation-specific family transcription factors in response to androgen, and thus, it aberrantly activates downstream oncogenes that play important roles in many biological processes, including cell proliferation, angiogenesis, and invasiveness.^40^, ^41^, ^42^ FGFR3–TACC3, in glioblastoma, is an example of tandem duplication generating a fusion protein that leads to the constitutively activated kinase signaling of FGFR3, and thereby, promotes cell proliferation and tumor progression.^43^ Another example is the tandem duplication resulting in the C2orf44–ALK fusion, which occurs within chromosome 2 in colorectal cancer, leading to the overexpression of ALK kinase.^44^ These examples showed that genes forming fusions can lose or gain functions, and importantly, these alterations significantly alter and contribute to tumor biology through several approaches. Hence, some have been identified as promising diagnostic markers and therapy targets.^14^ For instance, imatinib is administered to patients who carry the BCR/ABL fusion, and crizotinib to patients who carry the EML4–ALK fusion.^45^^,^^46^ Gene fusions represents a promising therapeutic opportunity as they target specific domains of both genes conforming the fusions. For example, imatinib and crizotinib target the proteins by preventing the phosphorylation of the tyrosine domain.^45^^,^^46^ Despite the effort that has been made to characterize gene fusions, the gene fusion panorama remains poorly unknown and it becomes more complex if other RNAs are involved.

## Long non-coding RNAs comprise gene fusions with promising functional implications in cancer

Up to now, the most complete compilation of gene fusions has been annotated in the FUSIONGDB repository, which reports 126,000 human gene fusions.^47^ Little is known about them and the biological role they play, however, due to their clinical relevance as some of them have been proved as effective biomarkers and therapeutic targets, it is worth further describing this complex and unknown layer of regulation in tumor biology.^47^ Nonetheless, most of the transcriptome is not protein-coding but non-coding transcripts, therefore, a substantial number of chromosomal rearrangements can be found in the non-coding regions, activating or silencing these genes.^48^

Interestingly, ncRNAs have been recently identified as another layer of complexity in the gene fusion landscape. In the last 20 years, lncRNAs were considered junk DNA, and now, they have been identified as part of these major chromosomal rearrangement events. In this regard, several questions arise. Is their function compromised when conforming fusions? Are they transcribed or translated? If so, can they contribute to the tumoral phenotype through gaining and losing functions likewise PCG fusions? Are lncRNA fusions potential biomarkers and targeted therapy?

**Figure 1:**
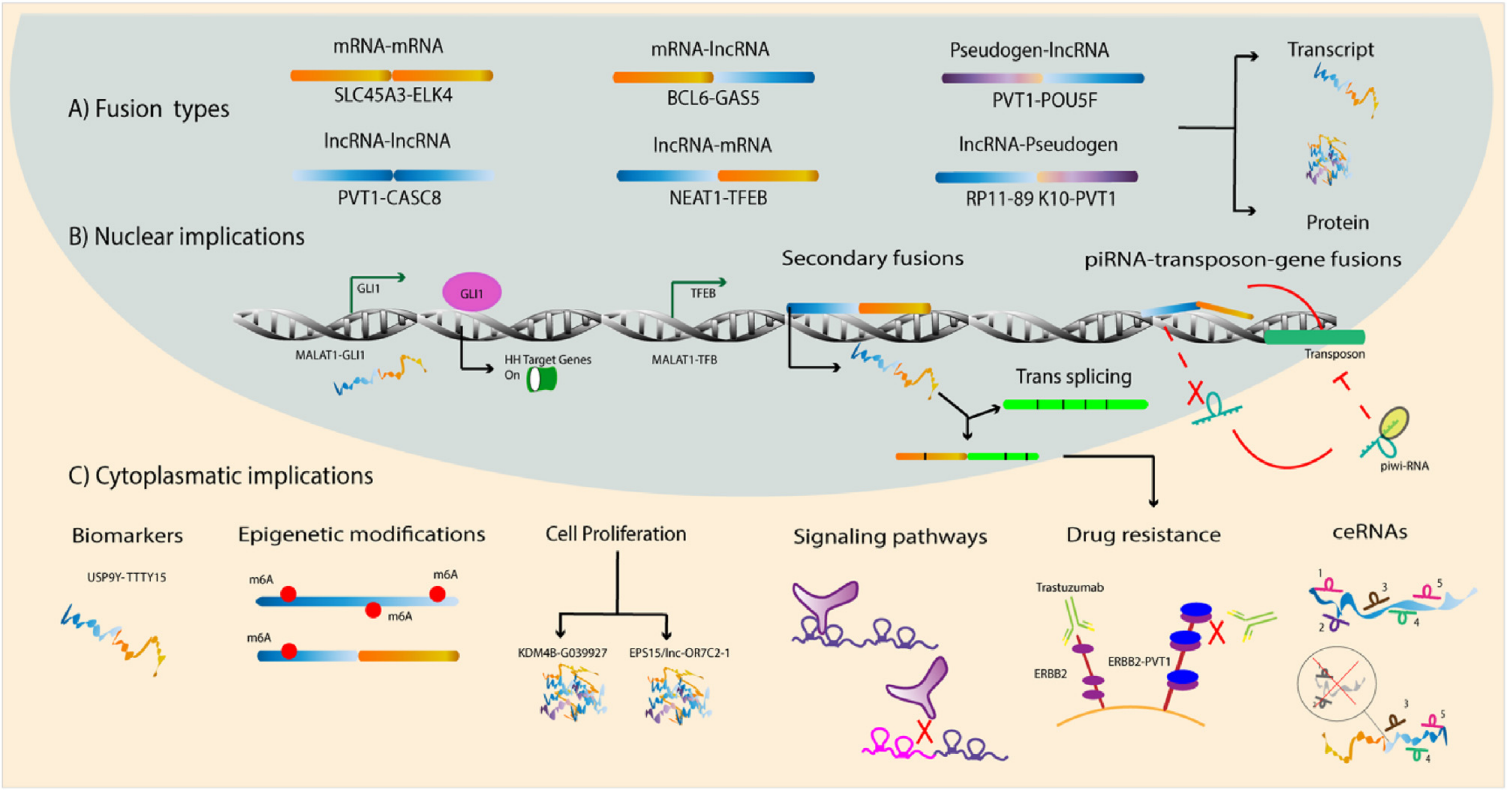
LncRNA fusions. **(A)** Types of lncRNA fusions based on promoter and sequence order which can produce either a functional transcript or a protein. **(B)** Nuclear implications of functional lncRNA fusions: promoter activation, signaling pathway activation, production of secondary functions, and generation of lncRNA fusions due to piwi-RNA dysregulation. **(C)** The cytoplasmatic implication of functional lncRNA fusions: highly and specifically expressed lncRNAs as potential biomarkers, alteration of epitranscriptomic markers, oncological phenotypes such as enhanced cell proliferation, disruption of signaling pathways, drug resistance, and alteration of ceRNA interactomes.

Canonical or most known gene fusions composed exclusively of PCG can gain or loss functions through several mechanisms. One way is aberrant activation or repression at the transcriptional level: TMPRSS2 fusion with ERG is a prime example as the first causes overexpression of the second gene through promoting its transcription.^40^ This example is representative when talking about lncRNA fusions as they are subject to the same regulation in which either the promoter of a lncRNA or a PCG can dictate the second gene function/regulation. There are three types of conformations based on the position of the lncRNA in the fusion: mRNA–lncRNA, lncRNA–mRNA, and lncRNA–lncRNA, as shown in Figure 1. In the first, the fusion is constituted by a lncRNA regulated by the PCG promoter. In the second, the PCG is regulated by the lncRNA region, and in the third one, as both genes are non-coding RNAs, the regulation depends on the lncRNA adjacent to the promoter.^6^ However, the order of the genes not only can determine gene fusion regulation at a genomic level but if transcribed, can determine the fate of the fusion gene, which can lead to two pathways: a functional transcript or a transcript translated to a functional protein. It is likely that gene fusions exclusively conformed by PCG or fusions in which the first gene is a PCG, the transcripts can be translated into proteins and have a deleterious effect. On the contrary, for fusions in which the first gene is a lncRNA or fusion lncRNA–lncRNA, it is reasonable to consider they do not achieve translation or are not even transcribed. Nonetheless, based on recent studies, there is evidence to point out non-coding RNA fusions as potential transcripts or even proteins with a biological role through loss/gain of interactions and functions.^49^ Interestingly, an additional type has been described; PCG and lncRNAs are not the only participants in gene fusion but pseudogenes as well. Originally, pseudogenes were considered junk DNA and now it is known that they lead to gene divergence playing an important role in cell evolution.^50^ Many of them even have introns and regulatory sequences acting as regulatory molecules of their parental genes, trans-acting elements, and microRNA decoys.^51^, ^52^, ^53^ However, what would be the implication of gene fusions confirmed by pseudogenes and lncRNAs? Likewise, in PCG–lncRNA fusions, pseudogenes fusions can have two arrangements: pseudogene–lncRNA which might undergo translation as some pseudogenes code for small proteins, or lncRNA–pseudogene, in which we would expect loss/gain of regulatory regions.^54^ Below are some examples, nonetheless, it is important to mention that our search for gene fusions with lncRNAs was made difficult by the lack of a homogeneous nomenclature.

Guo and collaborators described the landscape of gene fusions considering the classification based on the gene promoters and adding the PCG-exclusive gene fusions.^6^ From 28 cancer types of RNA-seq from the TCGA, they identified gene fusion events and the result showed that lncRNA forming fusions outnumber PCG fusions, ranking first. Interestingly, mRNA–lncRNA fusions were the majority compared with mRNA–mRNA, lncRNA–mRNA, and lncRNA–lncRNA. Authors attributed this as the promoters of mRNA promoters and their transcription machinery are more abundant.^6^ Notwithstanding, in our literature review, we found that lncRNA–mRNAs are more abundant than mRNA–lncRNA (87 lncRNA–mRNA *vs*. 25 mRNA–lncRNA) (Table 1). However, as explained before, it is worth analyzing how the altered structure and order of the gene fusions can have a functional effect (Fig. 1). As mRNA–lncRNA fusions are subject to regulation by the promoter of the PCG, they can be translated.^6^ For instance, the ORF-containing mRNA–lncRNA fusions, KDM4B–G03992, and EPS15L1–lncOR7C2 identified in breast cancer, produced proteins that could be detected by mass spectrometry. These gene fusions were selected to evaluate their function in SKBR3 cell lines, and it turned out they can promote cell proliferation compared with the parental genes overexpressed.^6^ These findings are also remarkable due to the role of the parental genes in cancer, for example, KDM4B is a hypoxia-inducible histone lysine demethylase that promotes DNA damage and genome instability through demethylating retrotransposons, especially in breast cancer.^6^

**Table 1:** LncRNAs as gene fusions.

Fusion gene	Type by combination	Functional implication	Cancer	Reference
MALAT-GLI1	lncRNA–mRNA	Sonic Hedgehog signaling pathway	Plexiform fibromyxoma	58
TRAF3-ENSG00000259717	mRNA–lncRNA		Metastatic head and neck cancer	85
PVT1-ENSG00000253288	lncRNA–lncRNA	Cetuximab & long overall survival	Metastatic head and neck cancer	85
FLBN-ENSG00000245384	mRNA–lncRNA	Cetuximab & long overall survival	Metastatic head and neck cancer	85
ENSG00000231669-MSN	lncRNA–mRNA	Cetuximab & long overall survival	Metastatic head and neck cancer	85
ENSG00000259446-RYR3	lncRNA–mRNA	Cetuximab & long overall survival	Metastatic head and neck cancer	85
ENSG00000231121-NAV3	lncRNA–mRNA	Cetuximab & long overall survival	Metastatic head and neck cancer	85
TRD1-PVT1	mRNA–lncRNA		Acute lymphoblastic leukemia	61
PVT1-UGCG	lncRNA–mRNA		Acute lymphoblastic leukemia	86
PVT1-ASAP1	lncRNA–mRNA		Acute myeloid leukemia	87
USP22-AF086125	mRNA–lncRNA		Acute myeloid leukemia	87
PVT1-IRF2BP2	lncRNA–mRNA		Acute myeloid leukemia	61
PVT1-SPRY2	lncRNA–mRNA		Acute myeloid leukemia	61
PVY1-PTP4A1	lncRNA–mRNA		Acute myeloid leukemia	61
PVT1-CASC8	lncRNA–lncRNA		Acute myeloid leukemia	61
PVT1-LINC00964	lncRNA–lncRNA		Acute myeloid leukemia	61
PVT1-IFRD1	lncRNA–mRNA		Acute myeloid leukemia	61
PVT1-UBB	lncRNA–mRNA		Acute myeloid leukemia	61
PVT1-NSMCE2	lncRNA–mRNA		Acute myeloid leukemia	61
PVT1-CXCR4	lncRNA–mRNA		Acute myeloid leukemia	61
PVT1-CASC21	lncRNA–lncRNA		Acute myeloid leukemia	61
PVT1-KB-1568E2	lncRNA–lncRNA		Acute myeloid leukemia	61
PVT1-RP11-89 K10	lncRNA–pseudogene			61
PVT1-MYC	lncRNA–mRNA		Acute myeloid leukemia, breast adenocarcinoma, colon cancer	61, 62, 63,65
PVT1-NFIL3	lncRNA–mRNA		Acute myeloid leukemia	61
PVT1-CCDC26	lncRNA–lncRNA		Acute myeloid leukemia	61
PVT1-CASC11	lncRNA–lncRNA		Acute myeloid leukemia	61
PVT1-LINC00824	lncRNA–lncRNA		Acute myeloid leukemia	61
HIST1H2BD-PVT1	mRNA–lncRNA		Acute myeloid Leukemia	61
CXCR4/PVT1	mRNA–lncRNA		Acute myeloid leukemia	61
RP11-89 K10-PVT1	Pseudogene–lncRNA		Acute myeloid leukemia	61
NSMCE2-PVT1	mRNA–lncRNA		Acute myeloid Leukemia	61
MDM2/PVT1	mRNA–lncRNA		Acute myeloid leukemia	61
PVT1/NSMCE2	lncRNA–mRNA		Acute myeloid leukemia	88
BF104016-NSMCE2	lncRNA–mRNA		Acute myeloid leukemia	88
BCL6/GAS5	mRNA–lncRNA		β-cell lymphoma	89
PVT1/IGK	lncRNA–mRNA		Burkitt lymphoma, multiple myeloma	90,66
PVT1/IGL	lncRNA–mRNA		Burkitt lymphoma, multiple myeloma	91,66
NEAT1-TFE3	lncRNA–mRNA		Renal cell carcinoma	92
MALAT1-TFEB	lncRNA–mRNA	Up-regulation of transcription factor	Sporadic renal cell carcinoma	93,94
PVT1/ARHGEF3	lncRNA–mRNA		Kidney adenocarcinoma	95
PVT1-ZCCHC7	lncRNA–mRNA		Diffuse large B-cell lymphoma	96
RFTN1-PVT1	mRNA–lncRNA		Diffuse large B-cell lymphoma	96
PVT1-NBEA	lncRNA–mRNA		Multiple myeloma	97
NBEA-PVT1	mRNA–lncRNA		Multiple myeloma	97
PVT1-WWOX	lncRNA–mRNA		Multiple myeloma	97
IGL-PVT1	mRNA–lncRNA		Multiple myeloma	66
PVT1-MERTK	lncRNA–mRNA		Adrenal adenocarcinoma	98
TMEM87B/PVT1	mRNA–lncRNA		Adrenal adenocarcinoma	98
KDM5B/PVT1	mRNA–lncRNA		Bladder transitional cell carcinoma	98
PVT1/EFNA1	lncRNA–mRNA		Bladder transitional cell carcinoma	98
GSDMC/PVT1	mRNA–lncRNA		Breast adenocarcinoma	99
AEBP2/PVT1	mRNA–lncRNA		Breast adenocarcinoma	98
GOLGA7/PVT1	mRNA–lncRNA		Breast adenocarcinoma	98
PVT1/AEBP2	lncRNA–mRNA		Breast adenocarcinoma	98
PVT1/C190RF57	lncRNA–mRNA		Breast adenocarcinoma	98
PVT1/CS	lncRNA–mRNA		Breast adenocarcinoma	98
PVT1/KHDRBS3	lncRNA–mRNA		Breast adenocarcinoma	98
PVT1/NABP2	lncRNA–mRNA		Breast adenocarcinoma	98
PVT1/PHF20L1	lncRNA–mRNA		Breast adenocarcinoma	98
PVT1/POU5F1B	lncRNA–pseudogene		Breast adenocarcinoma	98
PVT1/RGS22	lncRNA–mRNA		Breast adenocarcinoma	98
PVT1/SLC30A8	lncRNA–mRNA		Breast adenocarcinoma	98
TRPS1/PVT1	mRNA–lncRNA		Breast adenocarcinoma	98
PVT1/GSDMB	lncRNA–mRNA		Breast adenocarcinoma	64
CLPTM1L/PVT1	mRNA–lncRNA		Breast adenocarcinoma	100
KDM4B/G039927	mRNA–lncRNA	Proliferation and altered genes	Breast cancer	6
EPS15L1/lnc-OR7C2-1	mRNA–lncRNA	Proliferation and altered genes	Breast cancer	6
PVT1/CACNA2D2	lncRNA–mRNA		Colon adenocarcinoma	98
PVT1/MYC	lncRNA–mRNA		Colon adenocarcinoma	65
PVT1/ANXA13	lncRNA–mRNA		Esophagus adenocarcinoma	98
PVT1/FARP1	lncRNA–mRNA		Esophagus adenocarcinoma	98
PVT1/SNX31	lncRNA–mRNA		Esophagus adenocarcinoma	98
PVT1/TMEM67	lncRNA–mRNA		Esophagus adenocarcinoma	98
PVT1/MYC	lncRNA–mRNA		Esophagus adenocarcinoma	65
PVT1/BASP1	lncRNA–mRNA		Lung adenocarcinoma	98
PVT1/RNGTT	lncRNA–mRNA		Lung adenocarcinoma	98
PVT1/CHD7	lncRNA–mRNA		Lung small cell carcinoma	101
PVT1/CCNB1IP1	lncRNA–mRNA		Lung small cell carcinoma	102
PVT1/CLVS1	lncRNA–mRNA		Lung small cell carcinoma	102
PVT1/LY6H	lncRNA–mRNA		Lung small cell carcinoma	102
PVT1/MYH7	lncRNA–mRNA		Lung small cell carcinoma	102
PVT1/NOL4	lncRNA–mRNA		Lung small cell carcinoma	102
PVT1/SLC7A7	lncRNA–mRNA		Lung small cell carcinoma	102
PVT1/CHD7	lncRNA–mRNA		Lung small cell carcinoma	102
PVT1/AKT3	lncRNA–mRNA		Lung small cell carcinoma	102
PVT1/EYA1	lncRNA–mRNA		Lung small cell carcinoma	69
PVT1/CHD7	lncRNA–mRNA		Lung small cell carcinoma	103
PVT1-EXT1	lncRNA–mRNA		Lung squamous cell carcinoma	98
PVT1-RGS20	lncRNA–mRNA		Lung squamous cell carcinoma	98
RIMS2-PVT1	lncRNA–mRNA		Lung squamous cell carcinoma	98
ZNF521-PVT1	mRNA–lncRNA		Lung squamous cell carcinoma	98
PVT1-NDRG1	lncRNA–mRNA		Medulloblastoma	62
PVT1-ZC3H3	lncRNA–mRNA		Medulloblastoma	62
PVT1-LINC00964	lncRNA–lncRNA		Medulloblastoma	62
PVT1-COQ6	lncRNA–mRNA		Ovarian adenocarcinoma	98
SLC22A2/PVT1	mRNA–lncRNA		Ovarian adenocarcinoma	98
PVT1/EIF3E	lncRNA–mRNA		Prostate adenocarcinoma	98
PVT1/RNF139	lncRNA–mRNA		Prostate adenocarcinoma	98
PVT1/TNFRSF19	lncRNA–mRNA		Prostate adenocarcinoma	98
SLC30A4/PVT1	mRNA–lncRNA		Prostate adenocarcinoma	98
PVT1/SLC30A4	lncRNA–mRNA		Prostate adenocarcinoma	104
PVT1-TNFRSF19	lncRNA–mRNA		Prostate adenocarcinoma	104
RNF139-PVT1	mRNA–lncRNA		Prostate adenocarcinoma	104
PVT1-SMARCAD1	lncRNA–mRNA		Prostate adenocarcinoma	104
USP9Y-TTTY15	mRNA–lncRNA	Chinese populations	Prostate adenocarcinoma	105
MALAT-GLI1	lncRNA–mRNA	Sonic Hedgehog signaling pathway	Gastroblastoma	59
PVT1-PTK2	lncRNA–mRNA		Stomach adenocarcinoma	98
PVT1-QRSL1	lncRNA–mRNA		Stomach adenocarcinoma	98
PVT1-APIP	lncRNA–mRNA		Stomach adenocarcinoma	106
PVT1-ATE1	lncRNA–mRNA		Stomach adenocarcinoma	106
PVT1-PDHX	lncRNA–mRNA		Stomach adenocarcinoma	106
PDHX-PVT1	mRNA–lncRNA		Stomach adenocarcinoma	106
PVT1-PPAPDC1A	lncRNA–mRNA		Stomach adenocarcinoma	106
ERBB2-PVT1	mRNA–lncRNA		Uterine adenocarcinoma	98
PVT1-CFTR	lncRNA–mRNA		Uterine adenocarcinoma	98
PVT1-DPY19L4	lncRNA–mRNA		Uterine adenocarcinoma	98
PVT1-CD36	lncRNA–mRNA		Uterine adenocarcinoma	98
RP11–123O10.4–GRIP1	lncRNA–mRNA		Endometrial adenocarcinoma	107
RP11–444D3.1–SOX5	lncRNA–mRNA		Endometrial adenocarcinoma	107
RP11–680G10.1-GSE1	lncRNA–mRNA		Endometrial adenocarcinoma	107
RP11–96H19.1–RP11–446N19.1	lncRNA–lncRNA		Endometrial adenocarcinoma	107
NRIP1–AF127936.7	mRNA–lncRNA	Associated with stage	Endometrial adenocarcinoma	107

On the contrary, as lncRNA–mRNA fusions do not contain an ORF, it is reasonable to think they do not achieve translation, and hence, are degraded by mechanisms such as non-mediator decay or ubiquitinates leading to loss of function.^6^ Nonetheless, there may be another mechanism and it would be more interesting to think that these non-coding transcripts may be potentially functional. On one hand, it must be considered that some lncRNAs are translated to functional peptides such as LINC00266-1, which codes for a peptide named RBRP. This peptide induces colorectal cancer by interacting with the m^6^A reader IGF2BP1.^55^^,^^56^ Moreover, recent evidence shows that a gene fusion formed by two mRNAs SLC45A3 and ELK4, both known as oncogenes in prostate cancer, inhibits cell proliferation when the RNA fusion is silenced but not the protein parent genes. Remarkably, this fusion only represents 1% of the parental gene pool and it is enriched in the nuclear part of the cell fraction. Remarkably, this finding supports the idea that the function relies on the transcript, behaving as a lncRNA, the authors explained.^49^ This example represents a watershed in the role of gene fusions in tumor biology, particularly for lncRNAs as part of gene fusions because it demonstrates that despite lacking an ORF, these new structures generated by the genomic alteration can lead to gain and loss of function. In addition, it should not be ruled out translation to peptides for other cases since lncRNA–mRNA fusions can retain the ORF of the mRNA so translation remains feasible. However, a successful translation depends on the regulatory sequences of the lncRNA in the 3′ end, which can either promote or decoy translation.^57^ This reminds us of gene fusions like TMPRSS2-ERG, formed by only PCG, whose fate is dictated by the regulatory sequence of the first gene.^41^

Regarding the lncRNAs–mRNAs fusions, MALAT1–GLI1 is an example found in plexiform fibromyxoma and gastroblastoma. Strikingly, it is thought that the translocation of MALAT to GLI1 triggers overexpression of GLI1 protein. GLI1 is a key protein in the Sonic Hedgehog pathway, and therefore, this gene fusion favors the pathway activation.^58^^,^^59^ However, further study of these gene fusions is needed because it is not known how GLI1 is overexpressed.

A fascinating fact that we highlight in this review is that the lncRNA PVT1 is part of several gene fusions with mRNAs in numerous types of cancer, from leukemias to solid tumors such as breast, lung, prostate, esophagus, medulloblastoma, ovarian, stomach and uterine cancer (Table 1). In most cases, fusions involving PVT1 have only been identified and very few have been characterized. The oncogenic role of lncRNA PVT1 per se has been reported at several levels. For example, it has been reported to regulate the expression of genes involved in cell proliferation, migration, and invasion, through direct binding to the histone methyl transferase EZH2.^60^ In addition, it has been reported to have a ceRNA (competing endogenous RNA) role in competing with microRNAs for binding to mRNAs such as miR-152, miR-149, miR-186, and miR-195. It also binds to proteins, stabilizes MYC, and prevents its phosphorylation at threonine 58 and subsequent degradation by the proteasome.^60^ PVT1 function as a gene fusion is hardly known. In fact, PVT1 forms a fusion along MYC. As mentioned before, PVT1 wild type regulates MYC positively with tumoral downstream effects. Thereby, it is feasible that the PVT1–MYC fusion can have a potential oncogenic effect and could represent a possible biomarker due to its expression in several types of cancer such as colon, breast, and esophagus, leukemia, and medulloblastoma.^61^, ^62^, ^63^, ^64^, ^65^ However, there were other PVT1 fusions in which their gene partner shares genes across other types of cancer such as IGL, an immunoglobin already identified to form fusions with PCG such as MYC in multiple myeloma.^66^ It has been reported that patients carrying IGL fusions cannot be treated effectively with immunomodulatory drugs (IMiDs), implying that a fusion of IGL with PVT1 can similar consequences.^67^ In addition, PVT1 is the only gene reported so far that forms gene fusions with pseudogenes, such as RP11-89 K10 and POU5F1B in acute myeloid leukemia and breast cancer, respectively. The latter has been reported to promote tumorigenesis in gastric cancer and as it is located adjacent to MYC, therefore, it is reasonable that this fusion originates due to PVT1 and MYC association.^68^ PVT1 is an important lncRNA that plays several roles in cells, and that up to now, has the major number of gene partners when forming fusions. Altogether it suggests that the PVT1 gene fusions can have a functional role that significantly contributes to the tumoral phenotype.^67^

Our research has also found other shared PVT1 fusions; for instance, in leukemia, 26 gene fusions involving the lncRNA PVT1 were detected, and surprisingly, 15 of them with PVT1 as a 5′ partner shared the same breakpoint position. Astonishingly, the 5′PVT1/3′CCDC26 chimera was the only fusion detected in a panel of 23 leukemia cell lines.^69^ Nonetheless, chimeric transcripts involving PVT1 can also regulate the expression of yet unspecified target genes through “enhancer-like functions”.^70^ Therefore, gene fusions involving lncRNAs keep or even gain functional diversity from their parental genes.

Additionally, we hypothesize that some gene fusions may be generated through a transposon-PIWI interacting RNAs (piRNAs) dependent manner (Fig. 1). The mechanism consists as follows: piRNAs are small ncRNAs whose main function is transposon silencing, mainly in germline cells, to keep genome integrity.^71^ However, in cancer somatic cells, it has been described that the piRNA expression profile is dysregulated contributing to the disease phenotype.^72^ Once dysregulated, their regulation of transposons can be affected triggering genomic instability, and ultimately, creating gene fusions. In addition, there is a type of piRNA embedded in lncRNA sequences.^73^ Therefore, a potential feedback regulation could emerge through piRNAs creating lncRNAs fusions, and in turn, structural alterations of lncRNAs would deregulate piRNAs.

All the above gene fusions discussed so far mostly comprehend fusions originated as part of formation as genomic rearrangement, however, it is an enthralled fact that not all gene fusions arise from genomic alterations but can also be generated at the transcriptional level. Posttranscriptional modifications like splicing are responsible for the wide diversity of isoforms, and many of them are tissue-specific or functionally specific. At this level, the cross-strand chimeric RNA (cscRNA), a new class of gene fusions, has recently been described. These new gene fusions come from sense-antisense RNAs which are genes transcribed from both strands located close to each other, either by genomic region or three-dimensional chromosomal arrangements. Its proximity to each other facilitates the fusion of both genes by genomic rearrangements or trans-splicing.^74^ As of recent research, the functions of these rearrangements have not been described, however, it is reasonable to think that several cscRNAs are strongly associated with cancer, and by now, it is estimated that there are hundreds of them.^75^ Interestingly, lncRNAs might represent a great part of sense-antisense RNAs that form to cscRNAs since many well-known oncogenic lncRNAs are classified as natural antisense transcripts which are transcribed from the opposite strands of DNA that overlap with the sense strand and contribute to tumorigenesis.^76^ Furthermore, cscRNAs are not isolated evidence that gene fusions originate from the transcripts. In fact, Guo and collaborators identified and named “secondary fusions” generated from a previous one through cis or trans regulation.^6^ When a lncRNA can regulate a gene in cis or trans as an enhancer but is fused with another gene, whatever its nature is, the secondary fusion will be generated from a combination of the partner lncRNA gene and the gene that is under regulation. This is possible due to trans-activation events.^77^ Even more interesting is the fact that these secondary fusions represent one of the most complex levels of molecular heterogeneity that, indeed, can affect the tumoral phenotype. The same study also predicted that secondary gene fusions are formed by several druggable genes and target genes of current and novel treatments.^6^

All in all, cscRNAs and secondary gene fusions constitute a remarkable finding adding another layer of complexity that implies information not written in the but with potential biological functions. But how would those functions achieve? At the transcriptional level, secondary gene fusions can act through several mechanisms such as epigenetic modifications (*e.g.*, interactions in trans such as triple helix conformations with DNA,^78^ transcriptional interference, alternative splicing, and mRNA stabilization). These last three processes are of great relevance as it has been demonstrated that transcripts can positively regulate or negatively regulate other transcripts. For instance, natural antisense transcripts regulate their opposite-strand genes and it is feasible that when forming gene fusions, they can regulate antisense strands as their parental genes do.^79^

Lastly, as RNA editing is a fine-tuned regulatory process that RNAs undergo to achieve certain functions, it is interesting that any mechanisms orchestrated by epitranscriptomics may potentially be affected by lncRNA gene fusions.^80^ LncRNAs are subject to epigenetic modifications such as m^6^A, m^1^A, m^5^C, and pseudouridine.^80^ These modifications are highly dependent on sequence and structure and their functions are crucial to cell fate as they affect lncRNA's cellular localization and role in processes such as chromosome X silencing (*e.g.*, Xist silencing of chromosome X).^81^^,^^82^ The fact that mutations in MALAT1 can impair its m^6^A methylation, causing the impairment of the lncRNA function, is supporting the idea.^22^ Considering this example, it is reasonable to hypothesize that this regulation can be significantly altered when a lncRNA forms a gene fusion. This is worth mentioning for future study in the matter as it has not yet been considered until now.

Gene fusions described before involving lncRNA prove how wide the transcriptome diversity is and how little we know about their functions. To this matter, we have described the mechanisms through which the diversity of gene fusions may impact biological functions. However, the impact of these mechanisms on the evolution of tumor cells is still not clear. Are these fusions a consequence of genomic instability that together boosts a preexistent phenotype or are they able to drive the tumor cell to a particular phenotype like an advanced stage or a drug resistance condition resembling specific cases like the Philadelphia chromosome?^45^ There is still a lack of information to answer these questions but there is certainty about gene fusions involving lncRNAs generating cancer plasticity in both macro and microevolution contexts of the tumoral cell as explained by Mukherjee et al.^74^ On one hand, gene fusions are accumulated through structural changes with every cell cycle through genome instability because of transposable elements, mutations, copy number variations, and large rearrangements generating cancer progression (macroevolution events).^83^ A great example of it is the piRNA–transposon feedback postulated in the current work. On the other hand, especially in the context of lncRNAs as highly versatile and dynamic molecules, gene fusions can remodel the epigenetic, transcriptomic, and proteomic landscapes. In consequence, cellular fitness is enhanced by altering the phenotype that ultimately causes genomic structural alterations.^84^ However, the influence in the genomes is not exclusive of gene fusions generated by genomic changes but also to secondary fusions which is a remarkable fact as information not coded in the genome can drive structural alterations. Together, genomic-originated and secondary fusions set evolutionary feedback that could lead to clinically relevant scenarios in cancer.

## LncRNA gene fusions and the new therapeutic opportunity: current challenges

Of all the fusion lncRNAs we described in the previous section, it is really exciting to appreciate all the fusions in which PVT1 is involved, as it reminds us of the famous EML4–ALK gene fusion detected in anaplastic lymphoma and non-small cell lung cancer; despite its low prevalence, there are several drugs to inhibit its kinase activity, thus representing a watershed in therapeutics.^108^^,^^109^ These findings allow us to speculate that there is an emerging window of opportunity for further analysis of PVT1-generated fusions. However, the scenario becomes more lurid if PVT1 fusions are not translated into protein. Will these fusions be druggable? Notwithstanding, these gene fusions may be functional as additional or complementary biomarkers to existing ones. For instance, an interesting work reports that fusions consisting of lncRNAs (ENSG00000231669-MSN and ENSG00000231121-NAV3) were enriched in head and neck cancer patients with long progression-free survival.^85^ In contrast, Guo et al observed a general trend of better survival rates for patients with lower fusion events (although some did not reach significance).^6^ To be precise, the fusion KDM4B–G039927 was associated with poor outcomes in BRCA breast cancer patients.^6^ On the other hand, in endometrial cancer, the mRNA–lncRNA gene fusion NRIP1-AF127936.7 was found to be associated only with stage III disease, so it could have potential as a biomarker for staging classification.^107^ This topic, being so recent, still requires a significant amount of study.

The current challenges in identifying lncRNA fusion are the algorithms available, for example, Guo and coworkers used different annotation databases such as MiTranscriptome, NONCODE, and LNCipedia, with GENCODE used as a reference, and applying two efficient fusion calling algorithms Arriba and STAR-Fusion.^6^ Arriba has predicted that most of the fusions are originated from duplications or translocations and were the most found across the studies.^6^ Another important challenge is the study of the function of lncRNA fusions since this topic is still in its infancy. In this regard, numerous efforts strived to annotate lncRNA functions such as lncFunTk, lncLocator, iLoc-lncRNA, LncTar, AnnoLnc, and AnnoLnc2.^110^ It is worth highlighting that lncRNAs carry out their functions through various interactions, which can be affected by the generation of a gene fusion. In this context, AnnoLnc2 accounts for lncRNA–protein interactions based on both experimentally validated data and sequence-oriented prediction.^110^

It is widely described that lncRNAs are promiscuous genes with several versatile interactions with DNA, RNA, and protein.^13^ Therefore, it is reasonable to think that interactomes can vary greatly when lncRNAs form gene fusions. For instance, lncRNA fusions lead to the creation of new competitive endogenous RNAs as their interaction sites change. Furthermore, it has begun to elucidate the importance of lncRNAs in regulating signaling pathways through interaction with several proteins acting as scaffolds, guides, or translational repressors/activators. Hence, it is important to consider that loss and gain of interactions can considerably alter signaling pathway regulation, particularly in complex systems such as feedback and feedforward loops led by lncRNAs.^13^ In this regard, it has been demonstrated that lncRNAs boost drug resistance through deregulating signaling pathways by such mechanisms. In addition, secondary fusions predicted by Guo can diminish targeted therapy efficacy.^6^ If considering the latest factors, a bioinformatical and functional approach is proposed here to walk us through the understanding the role of lncRNA fusions in tumor biology. For instance, integration is required between the algorithms that predict gene fusions (exclusively PCG) such as FGviewer, and the ones that predict structure and function.^111^ Then, it is necessary to analyze the differences between the interactomes from a parental lncRNA and the gene fusion. Once achieved, it would be possible to proceed to evaluate functionality while working with data, in turn, it would bring us closer to knowing how and how impactful the emergent functional molecules work in cancer.

Another major challenge limiting the identification of fusion lncRNAs is the low expression of lncRNAs in general, but since they are so specific, they can be potential biomarkers.^105^ For instance, among the mRNA–lncRNA fusions, the USP9Y–TTTY15 gene fusion was found in prostate cancer in the Chinese population with a frequency of 21.4%. This fusion occurs between exon 3 of USP9Y and exon 3 of TTTY15, creating the transcript USP9Y–TTTY15, an mRNA–lncRNA fusion that has no ORF but has an effect in losing the function of the USP9Y gene. However, according to its high prevalence and the role of USP9Y as a protein like ubiquitin-specific proteases, the authors mention that it could have a relevant function.^105^

Furthermore, studies generally report lncRNA fusions in a range between 2 and 11,834 read counts in fusions like lnc-TMEM129-1-G059721 and MUTYH-G051064, in liver cancer and glioblastoma, respectively.^6^^,^^107^ This variation among read counts suggests that in case either of them is functional, they would greatly impact the tumoral phenotype; the first, despite its low expression, can remain functional while the second one could represent an advantage to the tumoral cell due to its high expression. In both scenarios, lncRNA fusions conserve high specificity not only across several cancer types but populations as very few fusions have been identified in more than one type of cancer. Hence, these characteristics could be determinant for a functional role in cancer as well as effective biomarkers.^49^

## Significance and future directions

All in all, studies suggest that fusion lncRNAs are highly relevant even though their identification and study are in their infancy. Therefore, one of the important limitations of this work is unknown information on the functions of fusion lncRNAs in cancer. However, there are no doubts that they are associated with disease development, and indeed prominently, they may also have great relevance in the treatment of cancer patients. In this regard, we set out to gather information on the identified fusion lncRNAs. Taken together, we found that these fusions, if they are mRNA–lncRNA, can be translated into proteins and have an oncogenic function such as KDM4B–G039927 and EPS15L1–lncOR7C2, through promoting cell proliferation.^6^ Whereas, if the fusion consists of mRNA–mRNA, it does not require translation to achieve functionality such as SLC45A3–ELK4.^49^ Regarding lncRNA–mRNA and lncRNA–lncRNA fusions, they are likely to remain in transcripts and the transcripts are likely to degrade or change the tertiary structure of the transcript and thus their interaction patterns and function change.^6^ In this review, we detected that PVT1 lncRNA forms a great variety of fusions with other lncRNAs and mRNAs in different types of cancer, but their functions are still unknown (Table 1). In conclusion, this review motivates us to conduct further research on lncRNA fusion and overcome the challenges and limitations (as well as the algorithms available for their identification and the experimental tools for their validation and to know their function), to clarify precisely the function of these fusions and their role in tumor development, as well as their role as therapeutic targets or biomarkers in cancer.

## Author contributions

D.S.M. and A.D.C.P. contributed to the conception of the study, wrote the original draft of the manuscript, collected intellectual critique information, revised the draft critically for important intellectual content, and reviewed and edited the manuscript. M.B.S.C. and F.I.P.R. contributed to the supervision of collected information and discussion of the data. R.G.R. contributed to guiding the writing, revising and editing of the manuscript.

## Conflict of interests

The authors declare that they have no known competing financial interests or personal relationships that could have appeared to influence the work reported in this paper.

## Funding

This research was funded by Instituto de Salud Pública, Universidad Veracruzana, Xalapa, Veracruz, México.
